# Identification of a female murder victim found in Burgenland, Austria in 1993

**DOI:** 10.1080/20961790.2021.1924425

**Published:** 2021-08-02

**Authors:** Christine Lehn, Andreas Rossmann, Matthias Graw, Gareth R. Davies

**Affiliations:** a Institute of Legal Medicine, Ludwig-Maximilians-University of Munich, Munich, Germany; b Laboratory for Stable Isotope Analytics, Isolab GmbH, Schweitenkirchen, Germany; c Faculty of Earth and Life Sciences, Vrije Universiteit Amsterdam, Amsterdam, The Netherlands

**Keywords:** Forensic sciences, forensic anthropology, human identification, provenance, stable isotope analysis, bio-elements, geo-elements

## Abstract

In 1993, the skeletal remains of a female corpse were found in Burgenland, Austria. Initial identification of the approximately 25–35-year-old female appeared impossible, but the case was reopened 23 years later. By applying biogeochemical isotope methods to her body tissues, the geographical origin of the unknown corpse could be predicted. The results of the C, N, S, H, Sr, and Pb isotope analyses suggested that the female did not originate from Europe and most likely spent her youth in the northern Caribbean. Using these findings, the police were able to identify the woman within 2 weeks. The female came from the Dominican Republic and resided in Austria for only a short period before she was murdered. This case shows that isotope biogeochemistry investigations can provide the police with crucial information that enables unknown persons to be identified.

**Keypoints**
- C-N-S-H and Sr-Pb isotope analyses were applied to human remains associated with a cold case.
- It was possible to determine the region of origin of the unknown deceased individual as the northern Caribbean.
- After 23 years, the murder victim was successfully identified.

## Introduction

Prosecution for murder is not time-limited and forensic methodologies like DNA analysis are constantly evolving, and “cold” murder cases can sometimes be solved and the perpetrators convicted even decades after the crime. One scientific method that is being increasingly used to answer various forensic questions is multi-isotope investigation. Using stable isotope analyses of the bio-elements and radiogenic isotopes on human remains, such as teeth, bones, hair, or nails, it is possible to reconstruct the life history of an individual [1–10]. This method is being used in forensics and archaeology to determine mobility during life and aid in the identification of deceased persons.

Cold cases are defined as previously unsolved criminal cases and are often subjected to new scientific methods with the hope that new results will help solve the case. The USA is a pioneer of cold case analysis, where the Federal Bureau of Investigation (FBI) established a cold case team as early as the mid-1990s. In Austria, the Federal Criminal Police Office has had a department for cold case investigations since 2010 and is responsible for management and coordination of national and international measures taken to investigate unsolved missing person and crime cases.

### Case background

In 1993, a partly skeletonised body of a female wrapped in plastic bags was found beneath bushes in a horse paddock in Burgenland, Austria, close to the Hungarian and Slovakian borders. The autopsy revealed that the female had died approximately 5–7 months before from a neck trauma. Her age was estimated to be 25–35 years. The police concluded that the deceased was a prostitute and originated from south-eastern Europe, Poland, or the former Yugoslavia. Despite extensive investigations, the female remained unidentified.

In 2016, the Austrian Federal Ministry of the Interior commissioned an isotopic investigation of the unknown female’s remains. The tissues available for analysis were a humerus and a femur.

### Elemental turnover times in bone tissue

The methodology used to determine changes in the geolocation of individuals is based on the analysis of isotope ratios of chemical elements in body tissues that are incorporated from food and drink.

To understand potential changes in the geographical residences of an individual, ideally tissue formed at different periods of a person’s lifetime are available to be studied [1, 4, 5]. Examples include teeth that develop during childhood and adolescence, tissue such as hair and nails that contains a relatively recent (weeks or months) isotopic signature, and bones with different rates of remodelling, such as rib or femur, that incorporate the elements over several years or decades before death. The present case was, however, limited to isotopic analysis of similar skeletal elements, the dense part (diaphysis) of a femur and a humerus. Analysis of a single skeletal element places limitations on data interpretation because any potential movement of the individual during their lifetime is impossible to establish. The signature of a specific tissue represents the integration of all elements taken up by the body while the relevant part of the bone was being formed and remodelled, in this case the compact material of the femur or humerus. Limb bones have dense structures with a relatively low rate of bone remodelling. Estimated turnover rates for compact bones in adults are ∼3% per year [11–13]. Given an age estimate of 25–35 years for the deceased female, the Pb-Sr and C-N-S-H isotope data of the femur and humerus bones represent an integrated record of her adolescence and adulthood. Depending on the actual age of the unknown deceased individual, the isotope data contain information about geographical residences or living conditions during the last 10–20 years of her life.

## Materials and methods

Initially, half a femur bone was provided by the Federal Criminal Police Office of Austria, but afterwards, doubts arose from the police officers if it was from the murder victim. A piece of humerus was provided that was undoubtedly from the deceased woman from Burgenland. Both bones appeared very light in colour and had likely been treated with hydrogen peroxide to remove the soft tissue. For the isotopic analytical investigations, the bone surfaces were ground away and bone pieces were sawn from the diaphysis. The bone pieces were crushed using a tungsten carbide ball mill (MM 301, Retsch GmbH, Haan, Germany).

### C-N-S-H stable isotope analyses

The crushed bone material was defatted with petroleum ether (40 °C–60 °C, p.a., Roth, Karlsruhe, Germany) for 3 h in a Soxhlet apparatus. Collagen was extracted following the method of Ambrose and Norr [14] and Longin [15]. Briefly, 500 mg of the defatted bone material was demineralised with 1 mol/L hydrochloric acid (30% p.a., AppliChem GmbH, Darmstadt, Germany) for 40 min at room temperature. After centrifugation and neutralization with demineralised water, the residue was gelatinized with 1 mmol/L hydrochloric acid (pH 3) for 16–18 h at 90 °C. The gelatine solution was pressure-filtered through a glass-microfiber disc and membrane filter (cellulose nitrate, 5 µm, Whatman AE 98; GE Healthcare, Buckinghamshire, UK). Subsequently, the filtered solution was freeze-dried. The dry collagen was used for stable isotope measurements of hydrogen, carbon, nitrogen, and sulphur with an isotope ratio-mass spectrometer (IR-MS).

For simultaneous analyses of C-N Sisotopes, 3.0 mg of the collagen samples were weighed into tin capsules (4 × 6 mm, IVA Analysentechnik, Meerbusch, Germany) in quadruplicate. For hydrogen isotope analyses, 150 µg were weighed into tin capsules in triplicate. In-house standards used for calibration were casein, porcine collagen, and bovine collagen. Bulk stable isotope ratios in the collagen samples were analysed at Isolab GmbH, Schweitenkirchen, Germany. The analytical details, including the values for the standards used, are reported in Lehn et al. [16].

### Sr and Pb isotope analyses

Powdered bone was weighed into pre-cleaned Teflon beakers (200 mg) and digested in 3 mL 14 mol/L HNO_3_ acid at 110 °C on a hot plate. The sample was then dried down on the hot plate at 100 °C. After being completely dried, the sample was dissolved in 2 mL 3 mol/L HNO_3_. The sample dissolution and low-blank chromatographic column chemistry methodologies used for Sr and Pb separation are described in full in Font et al. [17], where they were applied successfully to human teeth and hair. The acids used during the chemistry procedures were purified by sub-boiling distillation in Teflon from initial pro-analysis grade acids (Merck, Kenilworth, NJ, USA).

The Sr fractions were loaded onto Re filaments using a TaF_5_ activator to enhance ionization. Sr isotope ratios were determined on a Triton-plus Thermo Finnigan Thermal Ionisation Mass Spectrometer (TIMS; Waltham, MA, USA) instrument at the Vrije Universiteit Amsterdam. ^87^Sr/^86^Sr ratios were measured using a static multi-collection routine. An analysis consisted of 15 blocks of 10 cycles with an integration time of 8.1 s per cycle. ^87^Sr/^86^Sr and ^84^Sr/^86^Sr ratios were corrected for mass fractionation using an exponential law and ^86^Sr/^88^Sr ratio of 0.1194. Analyses of international standard NBS987 on load sizes of 100 ng were carried out to monitor performance and yielded average ^87^Sr/^86^Sr and ^84^Sr/^86^Sr ratios of 0.710239 ± 0.000005 (2SD) and 0.056491 ± 0.000003 (*n =* 15), respectively.

Lead isotopes were analysed on a Thermo Finnigan multicollector inductively coupled plasma mass spectrometer (MC-ICPMS). The samples were measured using a standard-sample bracketing method [18, 19] in a static multi-collection routine. NBS SRM-981 standard was used for bracketing and an in-house standard Pb solution (100 ppb CPI International) was used to check instrument performance during Pb isotope analysis. Standards were measured in 100 ppb solutions with the NBS SRM-981 standard values of Baker et al. [20]. Sample Pb concentrations were typically <100 ppb. Instrument blanks were analysed before and after each standard and sample, and the average of these two measurements was subtracted from each cycle before calculation of the Pb isotope ratios. Instrumental blanks were always <5 mV for ^208^Pb. Samples were introduced into the MC-ICPMS using a CetacAridus I desolvating nebuliser. The washout time between analyses was 3 min and the uptake time was 1 min. Five blocks of 20 cycles were measured with an integration time of 4 s per cycle. Levels of ^201^Hg were monitored during each analysis, but the signal was always <0.003 mV and therefore no corrections for ^204^Hg on ^204^Pb were applied. A session of Pb isotope measurements typically consisted of 10 samples, two in-house standards, 14 NBS SRM-981 standards, and 27 blank measurements. The long-term average Pb isotope data of a 100 ppb solution of the CPI International standard, including the values for the analytical session that produced data for this report, are reported in D’Imporzano et al. [21].

## Results

Stable isotope ratios for the bio-elements C, N, S, and H determined from the collagen are reported as *δ*^13^C_VPDB_, *δ*^15^N_AIR_, *δ*^34^S_VCDT_, and *δ*^2^H_VSMOW_ values. The isotopic results in the bone collagen samples of the unknown dead female from Burgenland are presented in Table 1. The Sr and Pb isotope data of the femur bone sample are presented in Table 2.

**Table 1.** C-N-S-H stable isotope values in bone collagen samples.

Sample	*δ*^13^C [‰ _VPDB_]	*δ*^15^N [‰ _AIR_]	*δ*^34^S [‰ _VCDT_]	*δ*^2^H [‰ _VSMOW_]
Femur bone collagen	−16.4 (−17.0)	11.8 (10.3)	9.4 (11.1)	−12 (−45)
Humerus bone collagen	−16.5 (−17.1)	11.7 (10.2)	10.0 (11.7)	−10 (−43)

In parentheses: bone collagen values converted to hair keratin values (according to [5]).

**Table 2.** Sr and Pb isotope composition of femur bone sample from the deceased. The errors are ± 2SE.

Sample	^87^Sr/^86^Sr	^206^Pb/^204^Pb	^207^Pb/^204^Pb	^208^Pb/^204^Pb	^206^Pb/^207^Pb
Femur bone	0.708457 ± 7	18.1217 ± 4	15.6001 ± 6	37.9199 ± 9	1.16162 ± 2

## Discussion

### C-N-S-H stable isotope data

Metabolic reactions in the body cause stable isotopic differences between the dietary and water input levels of various tissues [22–28]. The bone collagen data were converted into hair keratin values to use available databases. Mean isotopic offset between hair and bone collagen in modern humans (Δ_keratin-collagen_) is −0.6‰ for *δ*^13^C, −1.5‰ for *δ*^15^N, +1.7‰ for *δ*^34^S, and −33‰ for *δ*^2^H [5].

The converted collagen isotope values were compared with current C-N-S-H isotope data in our reference hair isotope database (a previous version published by Lehn et al. [5]) and with C-N isotope data in modern hair samples published by Hülsemann et al. [29]. The combined H-C-N-S isotope values from the two bone collagen samples are indistinguishable, confirming that the femur and humerus were from the same body.

**Figure 1.**
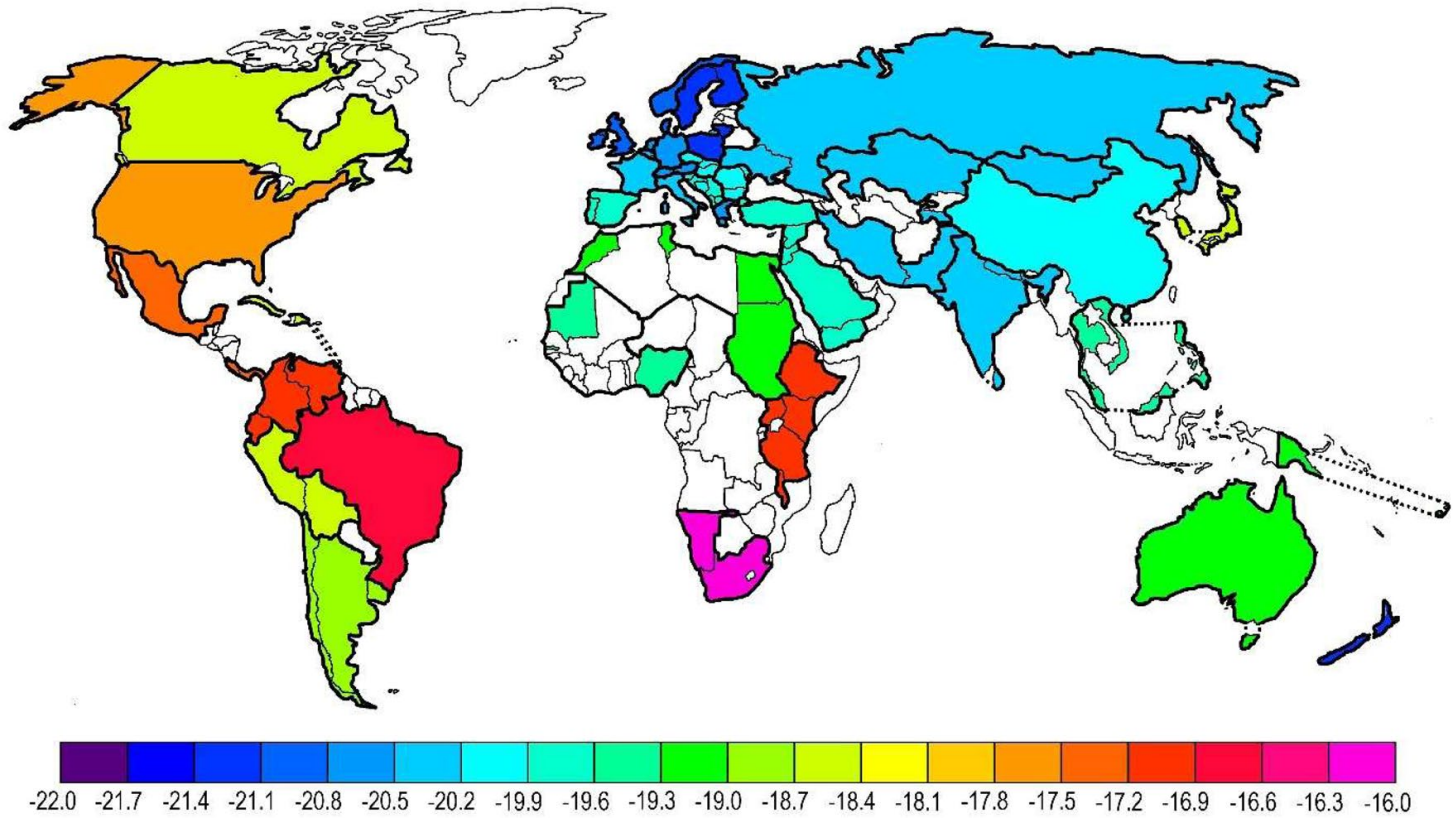
Global distribution of carbon isotope values in human hair samples of different geographical origin [29], reproduced with permission.

The carbon isotope values indicate a diet that contained a high proportion of C4 plants as a food source (maize, sugar cane, millet). Other cereals, rice, or potatoes probably played a subordinate role in the female’s diet. Such high values are very uncommon in human samples from Europe and Asia, but are more typical of samples from South and North America and parts of Africa (Figure 1).

**Figure 2.**
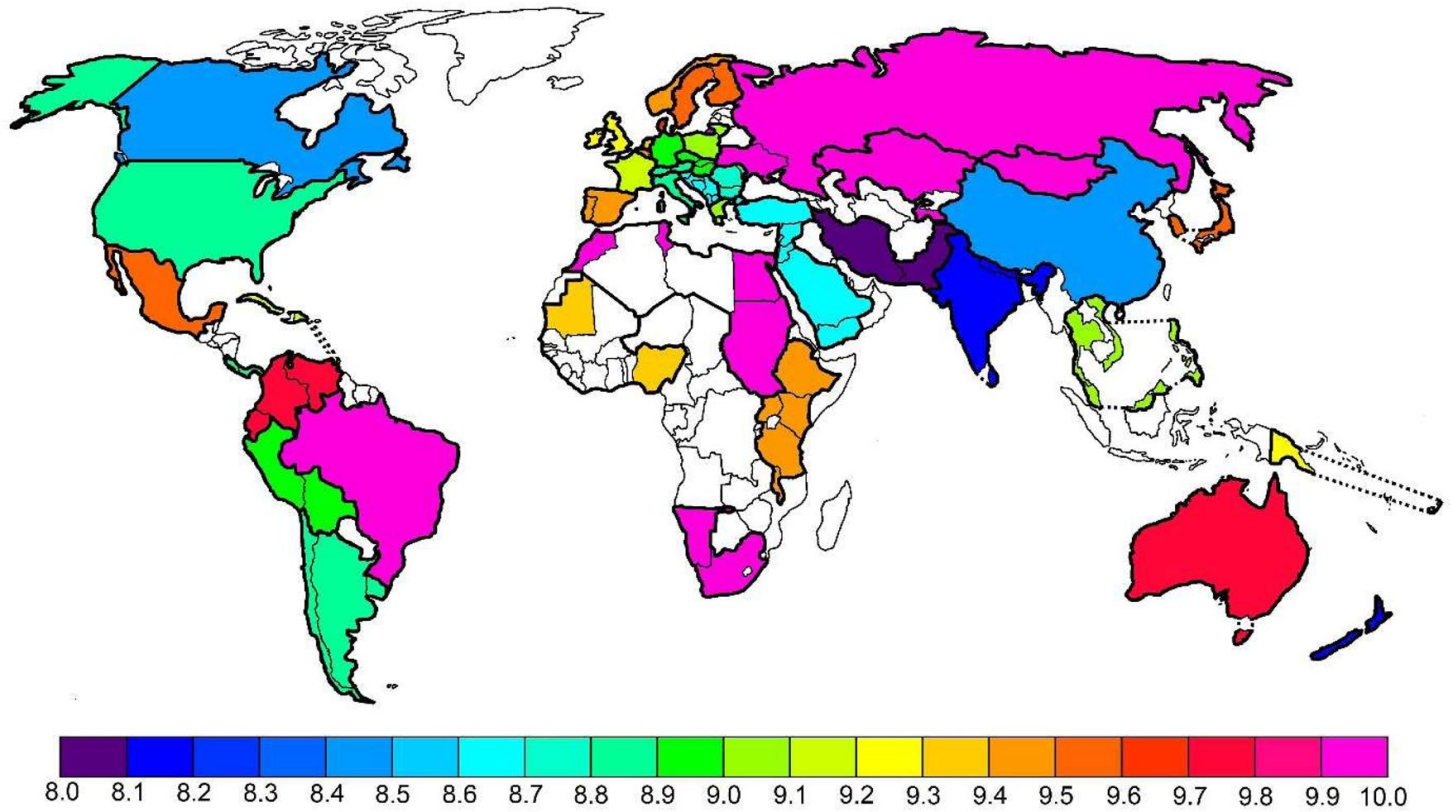
Global distribution of nitrogen isotope values in human hair samples of different geographical origin [29], reproduced with permission.

Both the nitrogen and sulphur isotope values in the femoral and humeral collagen were relatively high for modern human tissues (Figure 2) [5]. This suggests that the unidentified female had a diet largely consisting of marine protein, lived in a hot and dry climate with elevated nitrogen and sulphur isotope values, or both. She certainly did not have a vegetarian or vegan diet.

Such elevated N and S isotope values are very rare in hair samples from Europe. They are typically only seen in individuals who live immediately near the sea and mainly eat fish. Globally, such values are more common among people from Australia, New Zealand, the Philippines, and other countries where most cities are located close to the sea. In combination with the high carbon isotope values, however, these regions are inappropriate for the origin of the unidentified female in this case. The combined C-N-S isotope data from the female’s body tissues suggest that she lived near the sea in regions of South/Central America or Africa.

**Figure 3.**
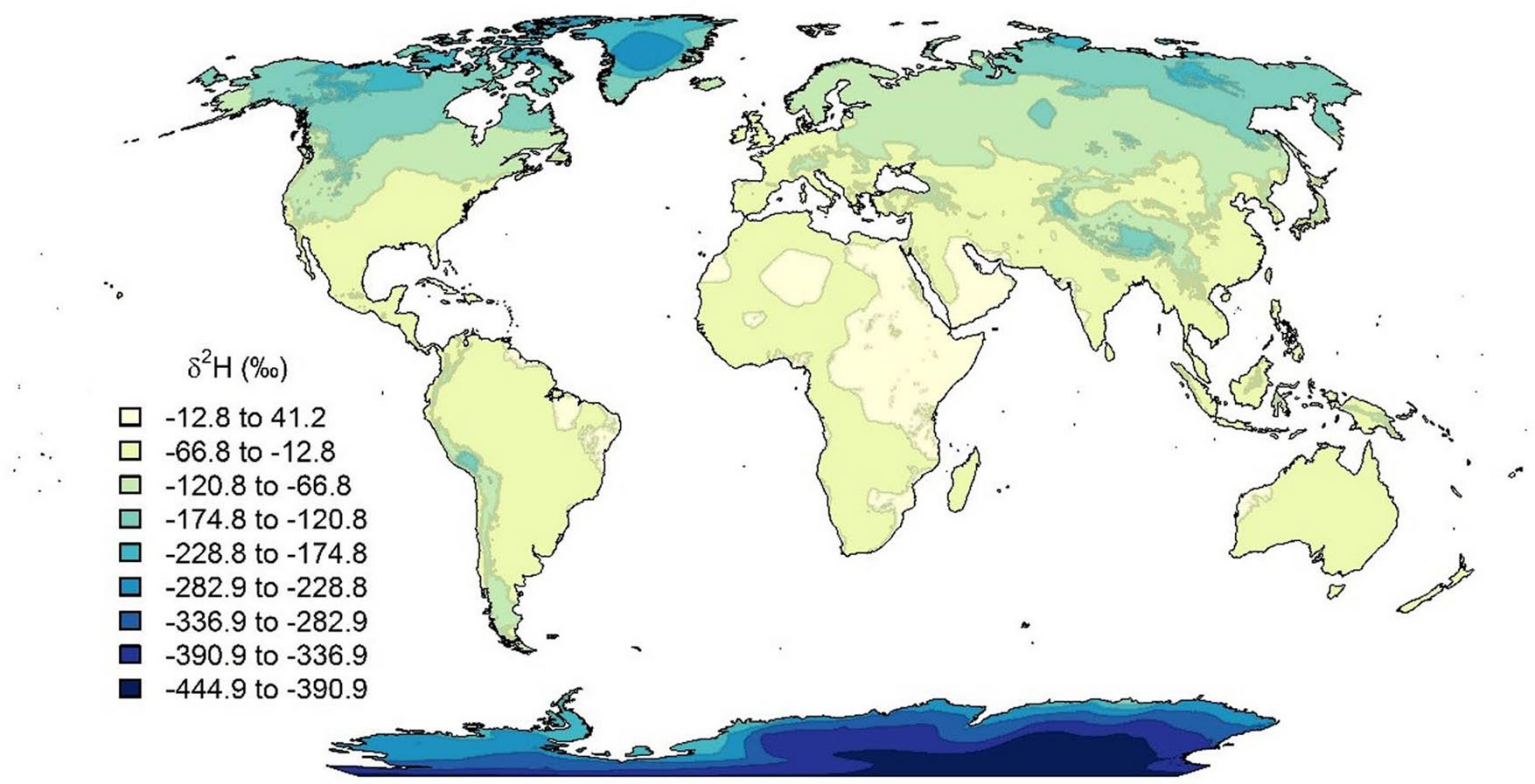
Global distribution of hydrogen isotope values in precipitation (Available at: https://wateriso.utah.edu/waterisotopes/media/IsoMaps/jpegs/h_Global/Hma_Global.jpg [Accessed 4 Jan 2021]) [36].

The isotope composition of hydrogen in human hair and collagen is mainly related to the local precipitation and tap water values [16, 30–35]. The hydrogen isotope values in the bone collagen samples here are comparatively high and suggest that the deceased mainly consumed food and drinking water from warm and dry climate zones (Figure 3). The values suggest that her residence was likely close to the sea in a low latitudinal region (<30°). From the *δ*^2^H values in the bone collagen, we can calculate an approximate mean precipitation *δ*^2^H value of −25‰. Such a value is incompatible with European values and is only seen in south-west Spain and Portugal, but the collagen C-N-S isotope data do not support such a region of origin. A corresponding climate zone can be found in north-western Brazil, the western and southern parts of Africa, the south-eastern USA, and coastal areas of Mexico and Central America, including the Caribbean Islands (Figure 3).

Statistical methods were used to determine possible geographic regions with a similar profile of C-N-S-H isotope values. The C-N-S-H isotope signature in the bone collagen samples of the unknown deceased were compared with the isotope data of our worldwide hair sample reference database and assigned to geographical regions by discriminant analysis. This method has proven to be a suitable tool for identifying the geographical origin of human remains [5]. The available reference hair database is constantly updated and contained 885 reference hair data from 75 different countries or regions at the time of data evaluation (Table 3). Not all countries worldwide have comprehensive coverage in the database and the number of reference hair samples varies greatly between regions. Although currently one of the best databases, it does not provide coverage for every area of the world and has low data resolution. Despite these limitations, multivariate analyses on predefined geographical groupings could potentially narrow down the region of origin of the unidentified female.

**Table 3.** Geographical groups of reference hairs from different continents and regions that are available for statistical evaluation of the isotope signatures of samples of unknown origin (status 29 Mar 2016, *N = *885). Mean ± 1σ.

Region	*n*	*δ*^13^C	*δ*^15^N	*δ*^34^S	*δ*^2^H
**Europe (*n =* 526)**					
Northern Germany	25	−20.9 ± 0.4	8.3 ± 0.6	7.4 ± 0.9	−74 ± 8
Southern Germany	74	−20.8 ± 0.4	8.6 ± 0.4	6.5 ± 0.8	−76 ± 9
Western Germany	27	−20.7 ± 0.4	8.4 ± 0.6	6.4 ± 0.5	−70 ± 9
Eastern Germany	23	−20.8 ± 0.3	8.2 ± 0.5	6.8 ± 0.8	−75 ± 8
Austria	23	−20.7 ± 0.4	8.4 ± 0.5	7.4 ± 1.3	−82 ± 11
Switzerland	21	−21.0 ± 0.4	8.5 ± 0.3	6.0 ± 0.8	−76 ± 11
Luxembourg	5	−20.2 ± 0.4	9.2 ± 0.4	6.7 ± 0.7	−63 ± 5
France	30	−20.2 ± 0.5	9.0 ± 0.7	7.5 ± 0.6	−63 ± 7
Spain	9	−19.8 ± 0.4	9.3 ± 0.7	8.0 ± 0.7	−60 ± 6
Belgium	6	−20.2 ± 0.5	9.2 ± 0.2	6.8 ± 0.7	−58 ± 6
Denmark	16	−21.0 ± 0.4	9.8 ± 0.4	7.7 ± 0.8	−68 ± 6
Norway	9	−21.1 ± 0.3	9.0 ± 0.4	8.5 ± 1.3	−71 ± 5
Sweden	4	−20.9 ± 0.6	9.2 ± 0.6	7.4 ± 0.2	−63 ± 10
Finland	3	−21.9 ± 0.8	9.4 ± 0.5	7.1 ± 0.9	−82 ± 15
Lithuania	19	−21.4 ± 0.7	9.1 ± 0.6	7.9 ± 0.8	−80 ± 6
Poland	29	−21.0 ± 0.5	8.9 ± 0.4	5.3 ± 0.6	−72 ± 10
Czech Republic/Slovakia	18	−20.5 ± 0.5	8.3 ± 0.6	5.8 ± 0.6	−75 ± 11
Ukraine	7	−20.9 ± 0.5	8.9 ± 0.6	6.4 ± 0.9	−84 ± 4
Hungary	6	−18.8 ± 0.6	7.7 ± 0.5	6.4 ± 0.6	−76 ± 5
Romania	28	−19.9 ± 0.7	8.2 ± 0.6	5.8 ± 0.6	−72 ± 7
Moldavia	5	−20.4 ± 0.5	8.9 ± 0.5	6.9 ± 0.3	−78 ± 9
Bulgaria	3	−20.3 ± 0.1	8.0 ± 0.1	5.8 ± 0.0	−72 ± 7
Greece/Cyprus	12	−20.2 ± 0.6	8.8 ± 0.6	8.1 ± 0.6	−61 ± 8
Italy	34	−20.4 ± 0.6	8.4 ± 0.7	6.3 ± 0.7	−72 ± 8
Serbia/Montenegro	9	−20.1 ± 0.4	8.4 ± 0.3	7.2 ± 0.2	−79 ± 9
Slovenia	2	−19.4 ± 0.5	8.4 ± 1.1	6.8 ± 0.3	−53
Netherlands	6	−20.7 ± 0.4	8.8 ± 0.5	6.5 ± 0.5	−76 ± 7
England	17	−21.3 ± 0.3	8.9 ± 0.4	6.3 ± 0.6	−73 ± 7
Ireland/Wales	13	−21.2 ± 0.2	8.8 ± 0.3	6.8 ± 1.0	−57 ± 5
Scotland	6	−21.6 ± 0.3	8.9 ± 0.5	6.3 ± 0.6	−67 ± 5
European Russia	37	−20.3 ± 0.7	9.5 ± 0.6	6.8 ± 0.8	−80 ± 9
**Middle/South America (*n =* 80)**					
Brazil	19	−16.1 ± 0.7	9.0 ± 0.6	9.6 ± 0.6	−57 ± 6
Costa Rica	19	−17.2 ± 1.1	8.8 ± 0.5	4.4 ± 1.5	−74 ± 7
Mexico	7	−18.1 ± 1.9	9.6 ± 0.5	6.3 ± 1.9	−71 ± 8
Peru	10	−18.6 ± 0.8	8.8 ± 0.3	5.7 ± 1.2	−89 ± 7
Chile	5	−19.2 ± 0.7	8.5 ± 0.8	9.2 ± 0.9	−67 ± 5
Cuba	4	−17.5 ± 0.5	9.8 ± 0.6	5.8 ± 1.1	−60 ± 9
Bolivia	5	−18.5 ± 1.1	9.0 ± 1.0	4.9 ± 0.7	−81 ± 9
Argentina	1	−18.0	9.4	7.8	−49
Martinique	7	−19.5 ± 1.1	8.9 ± 0.8	10.0 ± 1.2	−54 ± 10
Uruguay	1	−17.9	9.4	9.3	−
Guatemala	2	−15.3 ± 0.2	8.2 ± 0.2	5.4 ± 1.0	−73 ± 4
**USA/Canada (*n =* 33)**					
Illinois	8	−18.0 ± 0.6	8.2 ± 0.3	3.2 ± 0.8	−71 ± 7
Arizona	3	−17.4 ± 0.5	8.3 ± 0.3	2.6 ± 0.1	−71 ± 8
Michigan	3	−18.2 ± 0.6	8.2 ± 0.4	2.8 ± 0.1	−71 ± 5
Utah	3	−17.6 ± 0.7	8.4 ± 0.3	4.4 ± 1.3	−90 ± 1
California	1	−19.4	9.0	4.6	−78
Canada	15	−18.1 ± 0.5	8.3 ± 0.5	4.6 ± 1.3	−83 ± 6
**Australia/New Zealand (*n =* 20)**					
Australia	14	−19.3 ± 0.8	9.6 ± 0.7	11.9 ± 1.8	−49 ± 7
New Zealand	6	−20.9 ± 0.5	8.3 ± 0.4	13.0 ± 0.3	−64 ± 9
**Africa (*n =* 24)**					
Ethiopia	4	−15.6 ± 2.3	8.5 ± 1.3	10.4 ± 1.5	−36 ± 5
Nigeria	1	−19.1	8.5	8.3	−58
Morocco	2	−19.7 ± 0.4	9.0 ± 0.0	8.6 ± 0.4	−53 ± 16
Kenya	10	−17.7 ± 1.3	9.7 ± 0.8	8.8 ± 0.7	−42 ± 5
Namibia	5	−16.5 ± 1.0	10.4 ± 0.5	10.4 ± 0.7	−42 ± 3
Tanzania	2	−19.2 ± 0.7	9.2 ± 0.3	10.4 ± 2.4	−46 ± 2
**Asia (*n =* 202)**					
Turkey	21	−20.0 ± 0.4	8.4 ± 0.5	7.3 ± 0.4	−68 ± 7
Japan	29	−18.9 ± 0.4	8.8 ± 0.5	6.2 ± 1.0	−66 ± 8
Western China	12	−20.2 ± 1.1	7.5 ± 1.0	7.6 ± 0.5	−74 ± 9
Eastern China	27	−20.2 ± 1.4	7.8 ± 1.0	7.3 ± 1.4	−73 ± 7
Iran	8	−20.1 ± 0.4	7.9 ± 0.5	9.2 ± 0.5	−65 ± 5
India	23	−20.3 ± 0.8	8.0 ± 1.1	9.2 ± 1.4	−58 ± 8
Nepal	3	−20.8 ± 1.3	6.7 ± 0.7	6.5 ± 0.7	−76 ± 4
Indonesia/Malaysia	8	−20.0 ± 1.3	8.3 ± 0.5	8.0 ± 1.0	−67 ± 6
Sri Lanka	3	−20.4 ± 0.9	8.4 ± 0.4	9.0 ± 0.4	−59 ± 4
Saudi Arabia	1	−19.7	8.2	7.4	−67
Lebanon	10	−19.7 ± 0.7	8.4 ± 0.4	8.4 ± 0.7	−62 ± 7
Philippines	2	−19.3 ± 1.2	11.5 ± 0.1	8.7 ± 0.2	−70
Vietnam	1	−23.0	9.5	6.6	−76
Thailand	20	−19.5 ± 0.5	8.9 ± 0.5	7.4 ± 0.9	−61 ± 5
Korea	2	−18.7 ± 0.1	9.7 ± 0.4	8.2 ± 0.7	−58 ± 4
Mongolia	9	−20.1 ± 0.9	9.8 ± 1.9	6.3 ± 0.7	−87 ± 14
Pakistan	9	−20.4 ± 0.6	6.2 ± 1.8	3.8 ± 0.6	−61 ± 9
Kazakhstan	8	−20.0 ± 0.4	9.5 ± 0.4	6.0 ± 0.5	−73 ± 3
Russia East	6	−20.6 ± 0.4	10.7 ± 0.7	8.5 ± 0.8	−88 ± 7

All statistical analyses were conducted using IBM SPSS Statistics (Armonk, NY, USA). Discriminant analyses of the C-N-S-H isotope signature in the female’s remains were performed on the basis of regional groupings, including continents, groups of neighbouring countries, and geographical regions listed in Table 3. Furthermore, multivariate analyses were performed using different prior probabilities of the single groups (all groups equal, or computation depending on group size). From the results of discriminant analyses, Africa and Central/South America showed the highest probabilities of being the female’s region of origin. Within these geographical groups, the countries that yielded the highest probabilities were Namibia, Kenya, Brazil, and Martinique.

On the basis of the significance of the isotope values of the individual bio-elements, as well as the results of the statistical evaluations, the following conclusions about the dietary and geographical backgrounds of the unknown deceased from Burgenland are possible:

- The carbon isotope values indicate that for the majority of the female’s life she mainly fed on food produced in warm climatic regions. Her basic food contained a high proportion of C4 plants (maize, sugar cane, millet), which is atypical of most European diets.
- According to the nitrogen and sulphur isotope values, the female most likely lived in a coastal region and consumed a considerable amount of marine protein.
- Hydrogen isotope values indicate that the female probably came from a tropical or subtropical region. Most of Europe (except possibly the southern Iberian Peninsula) can be excluded as her place of geographical origin.
- Statistical evaluations of the integrated C-N-S-H isotope signature based on our reference hair database indicate the highest comparability to reference hair samples from Namibia or Kenya (“Africa”) or Brazil or Martinique (“Central/South America”).
- From the abovementioned findings of the C-N-S-H isotope data, we conclude the following regions have the highest likelihood of being the geographical origin of the female:

- Coastal regions of the southern African continent, such as Namibia, South Africa, Mozambique, Tanzania, or Kenya.
- Coastal regions of South America, most likely Brazil.
- Coastal regions of Central America, including the Caribbean islands (Greater and Lesser Antilles such as Cuba, Dominican Republic, Haiti, and Puerto Rico), and coastal cities of Central American countries (Mexico, Guatemala, El Salvador, Nicaragua, Costa Rica, and Panama).

Most importantly, the results of the stable isotopes of the bio-elements gave no indication for a European origin of the deceased. Of the European countries that were originally assumed as the origin of the female (Poland, Ukraine, Hungary, former Yugoslavia), none are considered compatible with the stable isotope data.

The isotope signatures in the female’s body tissues are relatively extreme, hence it can be assumed that she did not stay in Central Europe/Austria for an extended period of time to allow the collagen in the bone to turn over to a European diet. Given the expected elemental turnover rates, this would require several years. More precise statements about temporal and geographical changes within the last months or years of her life would only have been possible through additional isotope analytical examinations of her hair/rib bones, which unfortunately were not available for analysis.

### Sr and Pb isotope compositions

As both available bone samples would yield similar time-integrated information, Sr and Pb isotope analyses were only carried out on the femur. With only one analysis, any provenance conclusion would be based on the assumption that the female had not significantly changed location since her adolescence, and hence the Sr-Pb isotope signature would be controlled by a single region.

**Figure 4.**
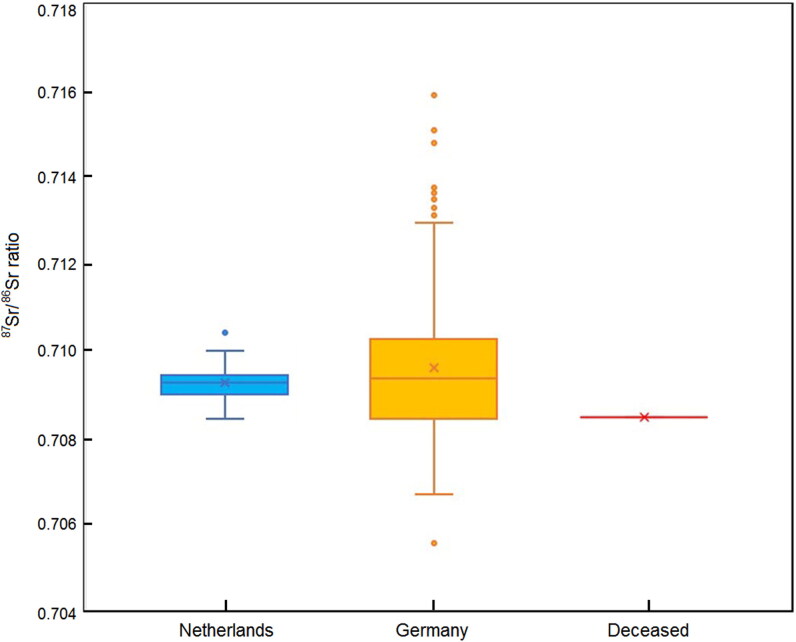
Sr isotope data in human enamel and environmental samples presented in a standard Boxplot figure: median and quartiles. Red is the femur from the deceased female. Dark yellow is the field from environmental samples from Germany. X = average, horizontal line=median. Outliers are shown as circles. Blue is bioavailable Sr from the Netherlands tooth database ([37] plus unpublished data). Sr isotope ratios in the German environment are significantly more variable than in the Netherlands bioavailable database because of i) the more diverse geology that includes volcanic regions such as the Eifel and old basement such as the Harz Mountains (0.707–0.720) and ii) the homogenisation of the environmental signature by the human body.

The measured ^87^Sr/^86^Sr ratio of the femur is relatively low compared with the bioavailable Sr that is characteristic of the sedimentary environments of most of north-western Europe, such as the river and glacial deposits (Figure 4). For example, the Sr isotope ratio falls outside 98% of the bioavailable data from the Netherlands and 75% of the environmental data from Germany. The latter range is expected to show substantially greater variation than the integrated bioavailable signature recorded within human tissue (see discussions in Plomp et al. [37, 38] and Kootker et al. [37, 38]). The relatively low Sr isotope ratio appears to rule out provenance of the deceased from regions dominated by sediments of fluvial, wind born (aeolian), and marine origin, and indicates that a significant proportion of the Sr in the female’s body was derived from either volcanic rock from the Earth’s mantle or limestone from regions of the Cretaceous, Jurassic, or Permian age (∼80–200, ∼250 Ma). This again rules out large parts of north-western Europe.

**Figure 5.**
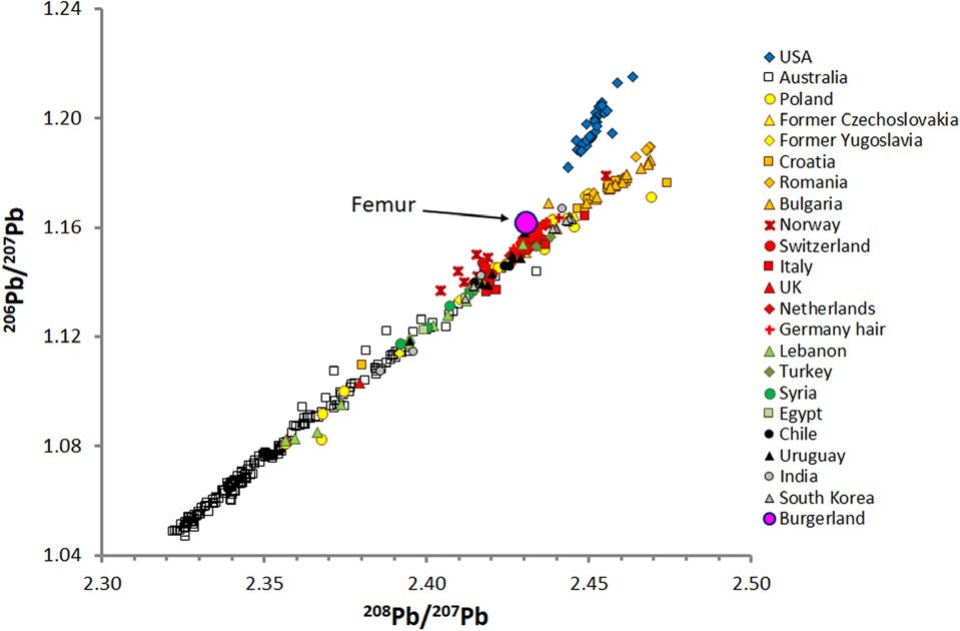
^206^Pb/^207^Pb *vs.* ^208^Pb/^207^Pb isotope ratios in the femur of an unknown female found in Burgenland and in tooth enamel samples from various countries in Europe, Asia and USA (see [10] and references therein). Note that USA samples have relatively high ^206^Pb/^207^Pb ratio at a given ^208^Pb/^207^Pb. The large purple-pink circle is sample of the deceased. Most available environmental Pb isotope data do not include ^204^Pb. The ^206^Pb/^207^Pb and ^208^Pb/^207^Pb figure is therefore used. Note that the error bars are smaller than the size of the symbols. The isotopic range of the limited Pb isotope data of German tooth enamel (*n* = 6) and hair samples (*n* = 13) from our unpublished data lie on the main data array with ^206^Pb/^207^Pb between 1.147 and 1.172. Additional US Pb isotope data from Air Force cadets can be found in Regan et al. [39].

Bioavailable Pb isotope data recorded in humans worldwide define a very strong positive correlation between the different Pb isotope ratios because of the similar geochemical behaviour of the parent isotopes (^235^U, ^238^U, ^232^Th) and the introduction of ancient Pb ores from Australia (Figure 5). The Pb isotope values of the deceased female are within the range recorded from human tissue from north-western Europe, such as parts of Germany and the Netherlands, or east and south-eastern Europe, such as Poland, Czechoslovakia, and Greece (Figure 5). The highest Pb isotope ratios shown in Figure 5 have greater variation away from the general linear relationship with some Eastern European regions (parts of Bulgaria, Poland, and Croatia) with relatively low ^206^Pb/^207^Pb ratios at a given ^208^Pb/^207^Pb ratio. Worldwide, the opposite signature, high ^206^Pb/^207^Pb ratios at a given ^208^Pb/^207^Pb ratio, is characteristic only of the USA. In the USA, atmospheric pollution from Pb added to gasoline as an anti-knock agent is characteristic of lead ores from the Mississippi valley region and leads to high ^206^Pb/^207^Pb at a given ^208^Pb/^207^Pb (Figure 5). The relatively small elevations in the ^208^Pb/^204^Pb, ^206^Pb/^207^Pb, and ^208^Pb/^207^Pb ratios for a given ^206^Pb/^204^Pb ratio in the female’s bone suggests the involvement of North American Pb. The relatively elevated ^206^Pb/^207^Pb ratio of the unidentified female is the key observation that caused us to suspect a USA connection. On the basis of the observation that mixing two components in a Pb-Pb isotope diagram always defines a straight line, a simple mass balance calculation indicates that <20% of the Pb in the female’s femur is derived from a source in the USA. The ^206^Pb/^207^Pb was elevated, but not sufficiently enough to indicate a person of American origin, and this region was therefore ruled out. The relatively elevated ^206^Pb/^207^Pb signal in the female’s bone is atypical of most of South America and almost all of Europe. There are a few samples recorded from Europe with similarly elevated ^206^Pb/^207^Pb signatures (Norway; Figure 5). However, according to the stable isotope data, these regions could be eliminated as possibilities.

The limited data available from the Caribbean (Cuba and parts of the Greater Antilles) show the influence of a North American Pb signal that was transported in air pollution. Prevailing winds in the northern Caribbean are, however, from the east. This suggests that the USA gasoline source in the region derives from North American gasoline used in Puerto Rico. Significantly, the characteristic Pb isotope signature is not present in most parts of South America. Coupled with the other isotopic data, the Pb isotope data suggest that the unidentified female is from a location where North American Pb contributes to the environment from pollution or trade. The Pb and C-N-S-H isotope data indicate a non-European provenance. On the basis of these observations, we proposed Mexico, the Bahamas, and the Greater Antilles (Cuba, Hispaniola, Puerto Rico, and Jamaica) as possible areas of origin for the deceased female. The Sr isotope results suggest that the unidentified deceased came from a region where the underlying geology contains a significant component of old limestone or volcanic rock. Moreover, the geology does contain a large proportion of Pre-Cambrian basement, as is characteristic of much of Africa and large parts of north and eastern South America. Sr isotope data from the Caribbean indicate that several islands have low ^87^Sr/^86^Sr ratios because of their volcanic origin [40, 41]. The data are incompatible with a provenance from the Caribbean islands that are predominantly composed of young limestones and have ^87^Sr/^86^Sr ratios >0.7085, but are compatible with Caribbean islands that are composed of volcanic rock, such as the northern Lesser Antilles or parts of the geologically older islands of the Greater Antilles that have limestones and a significant volcanic component.

## Conclusion

The isotope signatures of the elements in the femur and humerus of the 25–35-year-old female represent mean values of her average diet and environmental influences during the last 10 or 20 years of her life. No teeth, hair, or nails were available for investigation, hence it is not possible to make a detailed statement regarding her whereabouts in early childhood, nor give a temporal indication of possible changes in residence during the last months of her life.

The combined isotopic signal of the bio-elements (C-N-S-H) in the bone collagen of the deceased is rarely found in hair samples from Europe or Asia. The relatively high carbon, nitrogen, and sulphur isotope values are characteristic of a diet with large proportions of corn/sugar cane and marine protein. The high hydrogen isotope values indicate food and drinking water from a subtropical or tropical climate zone. The integrated isotopic signature is of a female most likely to have lived in coastal regions of subtropical or tropical climate zones of southern Africa, Central America, or northern South America.

The Sr and Pb isotope data indicate that the African continent can largely be excluded as her origin, as well as most regions of northern South America. Central America is the region with the highest probability of being her location of origin. From the Sr and Pb data, the northern Lesser Antilles or volcanic regions in the Greater Antilles, such as Cuba, Jamaica, Hispaniola, and Puerto Rico, are highlighted as most probable. According to the isotopic signature of the bio-elements, coastal regions of Central American countries, such as Mexico, Guatemala, El Salvador, Nicaragua, Costa Rica, or Panama, are also probable, especially particular areas that are exposed to some influence of North American lead in air pollution.

We concluded that the female could not have stayed in Central Europe/Austria for an extended period (>1 year), as a European influence on the isotope signature would begin to be discernible. The combined isotopic signature is atypical for Europe and indicates that the unknown deceased in Burgenland must have lived permanently in regions of Central America until shortly before her death.

In summary, we determined that the female must have lived most of her life in a warm climate zone with a diet rich in C4 plants and seafood. The results of the stable isotopes of the bio-elements gave no indication for a European origin. Furthermore, the Sr and Pb isotope data suggested that the deceased came from a region where the underlying geology does not contain a large proportion of Pre-Cambrian basement, which is characteristic of much of Africa and large parts of north and eastern South America. Pb isotope ratios were incompatible with most parts of Europe and much of Africa, as well as large parts of North and South America. Importantly, however, the Pb isotope data had relatively elevated ^208^Pb/^206^Pb and low ^206^Pb/^207^Pb ratios, indicating a component derived from North American Pb. The integrated isotope data were used to predict her origin from the northern Caribbean.

## Follow-up investigation by the police

On the basis of the conclusions of the isotope report, the cold case investigators from the Federal Criminal Police Office concentrated further investigations on the prostitution circles in the region of Austria where the body was found. They established the name of a missing prostitute from the Dominican Republic who was born in 1962, and obtained a comparative DNA profile from her sister *via* INTERPOL. This yielded a positive match. Within 2 weeks of receipt of the isotope report in 2016, the female was identified.

The deceased is known to have arrived in Burgenland in 1991 or 1992. She worked in a nearby brothel and disappeared overnight. The head of the cold case team reported that, “We had DNA from that time, but no comparative DNA.” On the basis of the regional situation, the police always believed that the deceased had come from Eastern Europe. Authorities in Hungary, Poland, the Czech Republic, and Slovakia, however, all failed to find a positive DNA match for the missing person.

The deceased female now has a name, and the investigators are attempting to identify the murderer in the Carinthia and Styria regions.
